# Pancreas-Brain Crosstalk

**DOI:** 10.3389/fnana.2021.691777

**Published:** 2021-07-20

**Authors:** Battuvshin Lkhagvasuren, Onanong Mee-inta, Zi-Wei Zhao, Tetsuya Hiramoto, Damdindorj Boldbaatar, Yu-Min Kuo

**Affiliations:** ^1^Brain Science Institute, Mongolian National University of Medical Sciences, Ulaanbaatar, Mongolia; ^2^Institute of Basic Medical Sciences, National Cheng Kung University College of Medicine, Tainan, Taiwan; ^3^Department of Psychosomatic Medicine, Fukuoka Hospital, National Hospital Organization, Fukuoka, Japan; ^4^Department of Cell Biology and Anatomy, National Cheng Kung University College of Medicine, Tainan, Taiwan

**Keywords:** pancreas, sympathetic nervous system, parasympathetic nervous system, intrapancreatic ganglia, enteropancreatic, circumventricular organs

## Abstract

The neural regulation of glucose homeostasis in normal and challenged conditions involves the modulation of pancreatic islet-cell function. Compromising the pancreas innervation causes islet autoimmunity in type 1 diabetes and islet cell dysfunction in type 2 diabetes. However, despite the richly innervated nature of the pancreas, islet innervation remains ill-defined. Here, we review the neuroanatomical and humoral basis of the cross-talk between the endocrine pancreas and autonomic and sensory neurons. Identifying the neurocircuitry and neurochemistry of the neuro-insular network would provide clues to neuromodulation-based approaches for the prevention and treatment of diabetes and obesity.

## Introduction

The pancreas maintains systemic metabolic homeostasis *via* exocrine and endocrine systems. Both systems receive enriched autonomic innervations (Tang et al., 2018a). Retrograde tracing studies have identified two distinct nerve fibers including the vagus nerve (parasympathetic) and the spinal nerve (sympathetic) innervating the pancreatic islets. Both of them can be further divided into two distinct types of nerve fibers: afferent fibers conducting sensory information from the pancreas to the central nervous system and efferent fibers conveying motor command from the central nervous system to the pancreas. Interestingly, the pancreas has its own intrinsic neurons gathered in the intrapancreatic ganglia scattered throughout the pancreas (Tang et al., 2018b), while their nerve fibers form the neuro-insular networks with specific nervous plexuses in the islets. However, exactly how the brain circuits regulate the endocrine functions of the pancreas is not well understood. For example, the main mediator responsible for glucose metabolism in the afferent nervous system is ill-defined and the structure and function of neurons in the spinal cord and medulla that project, directly or indirectly, to the pancreas are not identified. Furthermore, projections from the enteric nervous system in the gastrointestinal tract to the pancreatic intrinsic network are even less clear.

In addition to the neural pathways, the functions of the endocrine pancreas are regulated by humoral pathways from the circumventricular organs and peripheral chemoreceptors. The circumventricular organs, containing sensory neurons and tanycytes that can sense glucose in the blood, play an important role in maintaining systemic metabolic homeostasis (Verberne et al., 2014; Röder et al., 2016). However, the signal transduction and functional circuit in the higher-order brain centers are scarcely defined. The pancreatic islets consist of five major endocrine cell types, including α cells (~35%), δ cells (~55%), γ cells (~10%), γ/PP cells (~5%), and ε cells (~1%; Brissova et al., 2005; Cabrera et al., 2006; Blodgett et al., 2015). Besides β cells, other cell types in the islet also play roles in islet endocrine functions, insulin resistance, and pathogenesis of diabetes mellitus (Röder et al., 2016). Here, we review literature that provides empirical evidence showing cross-talk between the central nervous system and the pancreas.

## Extrapancreatic Nervous Division

### Sensory Innervation

#### Sensory Afferent Fibers of the Vagus Nerve (Parasympathetic Afferent Pathway)

**Figure 1:**
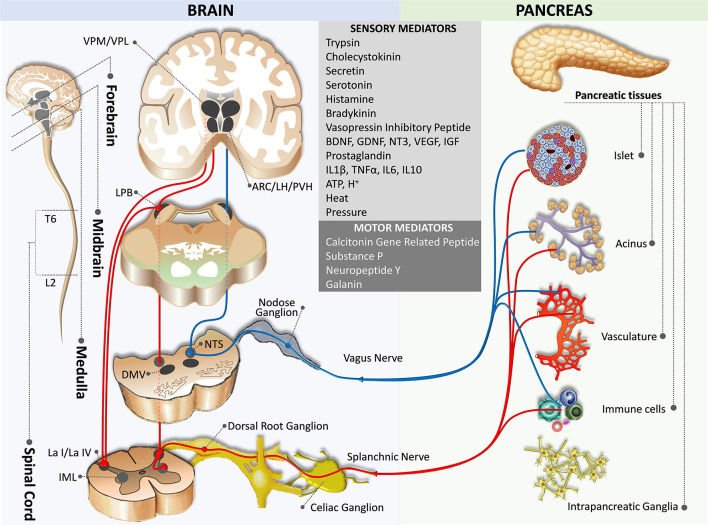
Sensory innervation from the pancreas to the brain. The blue pathway marks the parasympathetic afferent pathway, while the red pathway indicates the sympathetic afferent pathway. ARC,arcuate nucleus of the hypothalamus; DMV, dorsal motor nucleus of the vagus; IML, intermediolateral nucleus of the spinal cord; L2, spinal cord lumbar 2 segment; La I/La IV, lamina I and IV of the spinal cord gray matter; LH, lateral hypothalamic nucleus; PB, parabrachial nucleus; NTS, nucleus of the tractus solitaries; PVH, paraventricular hypothalamic nucleus; T6, spinal cord thoracic 6 segment; VPM/VPL, the ventral posteromedial/posterolateral nuclei of the thalamus.

The sensory afferent fibers of the vagus nerve originating from the pseudounipolar sensory neurons in the bilateral nodose ganglia (the inferior ganglia of the vagus nerve) are either small-diameter myelinated Aδ or unmyelinated C fibers. The afferent fibers travel with efferent fibers, contribute to the celiac plexus, and continue to follow blood vessels to reach the pancreas (Figure 1). Tracing studies on rodents demonstrated that the afferent fibers project from the blood vessels, ducts, acini, islets, and intrapancreatic ganglia, where they end freely within the pericapillary space in the pancreas without building neural synapses (Sharkey and Williams, 1983; Rinaman and Miselis, 1987; Neuhuber, 1989; Won et al., 1998; Love et al., 2007). However, human islets are less innervated by these neurons compared with rodent islets (Rodriguez-Diaz et al., 2011). The pancreas is abundant in parasympathetic afferent fibers which are involved in sensing chemical (e.g., trypsin, cholecystokinin, secretin, arginine vasopressin, serotonin, histamine, bradykinin, growth factors, prostaglandins, cytokines, adenosine triphosphate, acidity) and mechanical (e.g., heat and pressure) signals through different types of receptors and ion channels including the transient receptor potential cation channel vanilloid 1 (TRPV1) to control metabolic homeostasis, inflammation, and pain (Ahrén et al., 1986, 1987a; Kirkwood et al., 1999; Ahrén, 2000; Razavi et al., 2006; Schloithe et al., 2008; Barreto and Saccone, 2012). Interestingly, most of these sensory afferent neurons are also known to produce neuropeptides including substance P and calcitonin gene-related peptide (CGRP) to regulate inflammatory processes and mechanosensation (Sharkey et al., 1984; Won et al., 1998; Gram et al., 2007; Fasanella et al., 2008).

The specific role of the sensory afferent fibers of the vagus nerve in the exocrine and endocrine pancreas is not well described. Surgical- and chemical-related denervation have been used to study the function of the vagus nerve on this topic, although current literature has yielded conflicting results. Capsaicin at high doses acts as an excitatory neurotoxin *via* activation of TRPV1, which is expressed by most unmyelinated sensory neurons and some small-diameter myelinated sensory neurons (Caterina et al., 1997; Newson et al., 2014). Capsaicin-induced ablation of the vagus nerve had either no effect or a positive effect on insulin secretion (Karlsson and Ahrén, 1992; Karlsson et al., 1994; Panchal et al., 2018). On the contrary, a pancreatic vagotomy does not change insulin secretion (Imai et al., 2008). Glucagon secretion is also inconsistently affected by vagotomy (Karlsson and Ahrén, 1992; Jaworek et al., 1997; Plamboeck et al., 2013). Recent studies suggest that stimulations of the afferent or efferent fibers of the vagus nerve may exert differential responses in blood levels of glucose, insulin, and glucagon (Meyers et al., 2016; Payne et al., 2020). It has been demonstrated that electrical stimulation to the afferent fibers, but not the efferent fibers of the vagus nerve at low frequency (15 Hz) significantly increases levels of glucose and glucagon, while high frequency (40 kHz) stimulation lowers glucagon secretion (Payne et al., 2020). However, neither stimulations induce changes in insulin levels. Finally, a recent study suggests that vagal afferents express Phox2b, substance P, CGRP, and 5-hydroxytryptamine (5-HT) receptor type 3, which can sense serotonin in the pancreatic islets and terminate in the commissural nucleus of the tractus solitarius (Makhmutova et al., 2021).

#### Sensory Afferent Fibers of the Spinal Nerve (Sympathetic Afferent Pathway)

Sympathetic afferent fibers consist of small-diameter Aδ and C fibers originating from the pseudounipolar neurons in the dorsal root ganglia (DRG) at thoracic 6 (T6) – lumbar 2 (L2) segmental levels of the spinal cord. These afferent fibers transmit information from the pancreas to the spinal cord through the splanchnic nerves and celiac plexus and terminate monosynaptically in lamina I and IV in the dorsal horn at the same spinal segment (Renehan et al., 1995; Won et al., 1998; Andrew and Craig, 2001a,b; Buijs et al., 2001; Wilson et al., 2002; Furness, 2006; Lindsay et al., 2006; Figure 1). Neurons in the lamina I and IV project to sympathetic preganglionic neurons in the intermediolateral column of the spinal cord, thus forming a spino-spinal loop for somato-autonomic reflexes (Su et al., 1987; Craig, 1993).

Similar to parasympathetic afferent nerves, sympathetic afferent nerves transmit sensory signals from the pancreatic vasculature, acini, islets, and the intrapancreatic ganglia through the perivascular route. These fibers, expressing TRPV1 channels and producing substance P, CGRP, and neuropeptide Y (NPY), are involved in sensing both chemical and mechanical signals (Sharkey and Williams, 1983; Sharkey et al., 1984; Rinaman and Miselis, 1987; Su et al., 1987; Won et al., 1998; Razavi et al., 2006; Fasanella et al., 2008). Although little is known about the specific role of the nerves in the endocrine and exocrine functions of the pancreas, it is suggested that both parasympathetic and sympathetic afferent nerves expressing TRPV1 channels are involved in the inflammatory processes (Nathan et al., 2002). In contrast to nodose ganglion neurons, DRG neurons are high-threshold mechanoreceptors that are activated in response to noxious mechanical stimuli and play an important role in pain sensation and pancreatitis (Grundy, 2002; Liu et al., 2011). DRG neuron afferent fibers from the pancreas have been found to send collaterals to the celiac plexuses and sympathetic chain ganglia innervating the gut (Sharkey, 1987; Maggi and Meli, 1988; Maggi, 1990).

### Central Integration

#### Afferent Circuits

In general, signals on peripheral conditions are delivered to the Lamina I neurons through the somatosensory fibers of the DRG neurons, which then relay to the thalamic neurons and eventually project to the insular and somatosensory cortices *via* the spinothalamocortical pathway (Craig, 1996). However, Lamina I neurons also receive afferents from visceral organs and send collateral axons to the nucleus tractus solitarius (NTS), the dorsal motor nucleus of the vagus (DMV), and the caudal ventrolateral medulla (Neuhuber, 1989; Du and Zhou, 1990; Esteves et al., 1993; Craig, 1995; Renehan et al., 1995; Pan et al., 1999; Gamboa-Esteves et al., 2001; Fasanella et al., 2008; Figure 1). Furthermore, although direct projections between the pancreas and the brain through the nodose ganglia neurons have not been identified in humans, studies on rodents suggest that nodose ganglia neurons project to the NTS (medial, intermediate, parvicellular, and commissural NTS subnuclei) and the DMV, of which the latter is associated with the parasympathetic efferent pathway to the pancreas (Loewy and Haxhiu, 1993; Jansen et al., 1997; Streefland et al., 1998; Rosario et al., 2016; Figure 1). Retrograde transsynaptic tracing studies validate the aforementioned brain regions, together with other regions receiving afferent inputs from the pancreas. These include the caudal ventrolateral medulla, the locus coereleus, and the parabrachial nucleus (PB; Coleman and Hummel, 1969; Zhang et al., 1994; Williams and Elmquist, 2012; Meek et al., 2016).

NTS is located in the caudal medulla next to the DMV and the area postrema in the dorsal medulla. Together, these three nuclei are collectively called the dorsal vagal complex. The NTS receives sensory information from the entire gut on food composition and volume, condition of the lumen, enzymes, and peptide hormones through the vagus nerve (parasympathetic afferent fibers), sensory information from the aortic body and carotid body through the glossopharyngeal nerve, sensory information from the taste organs through the facial nerve, sensory information from the auditory organs through the vagus nerve, and modulatory information from the upper brain centers (Herbert and Saper, 1990; Larsen and Kristensen, 1997; Buijs et al., 2001; Niebergall-Roth and Singer, 2001; Barrera et al., 2011). The sensory representation of abdominal organs in the NTS has some distinct and overlapping areas (Hamilton and Norgren, 1984). After integration of the incoming information with other neuroendocrine signals, the NTS relays to a large number of brain regions including the DMV, the area postrema, the caudal ventrolateral medulla, the medullary reticular formation, the PB, the locus coeruleus, the paraventricular nucleus of the hypothalamus, the bed nucleus of stria terminalis, and the central nucleus of the amygdala (Fox and Powley, 1986; Neuhuber, 1989).

The PB, a homeostatic afferent integration center also receives projections from Lamina I, which then projects to the periaqueductal gray, a mesencephalic homeostatic motor center, thus forming a spino-tegmento-spinal loop (Westlund and Craig, 1996; Swanson, 2000). The PB, especially the lateral PB nucleus is one of the relay centers for ascending visceral sensory signals from the NTS to the hypothalamus (the ventromedial nucleus, the arcuate nucleus, the lateral hypothalamic nucleus, and the paraventricular nucleus), the amygdala, the thalamus, and the medulla (Sakumoto et al., 1978; Saper and Loewy, 1980). The projections between the PB and the complex network of the hypothalamic nuclei are responsible for the autonomic regulation of energy and metabolic homeostasis. The hypothalamus sends polysynaptic efferent projections to the peripheral organs, thus forming a spino-hypothalamo-spinal loop.

Tracing studies suggest that the PB projection originates in the portion of the NTS (caudal ventromedial nucleus) and the area postrema receiving visceral vagal afferents from the pancreas (Loewy and Burton, 1978; Herbert and Saper, 1990; Rosario et al., 2016). In addition to the ascending projection between the NTS and PB, there is a descending projection from the PB to the ventral part of the NTS through the Probst’s bundle (Saper and Loewy, 1980).

#### Efferent Circuits

**Figure 2:**
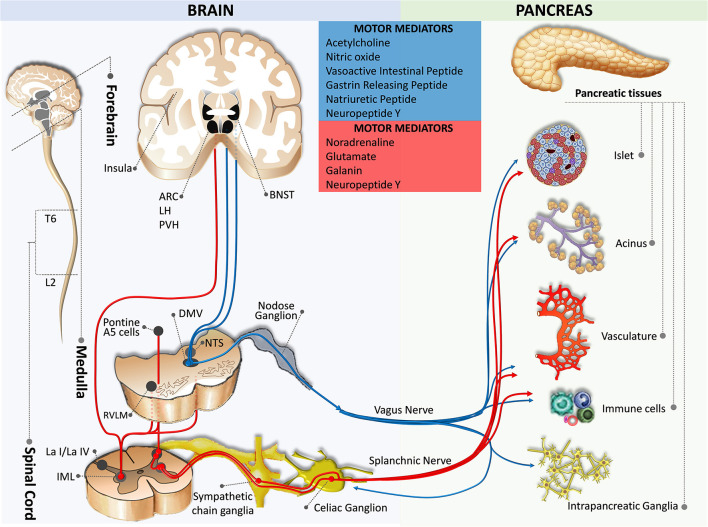
Motor innervation from the brain to the pancreas. The blue pathway marks the parasympathetic efferent pathway, while the red pathway indicates the sympathetic efferent pathway. ARC, arcuate nucleus of the hypothalamus; BNST, bed nucleus of the stria terminalis; DMV, dorsal motor nucleus of the vagus; IML, intermediolateral nucleus of the spinal cord; L2, spinal cord lumbar 2 segment; La I/La IV, lamina I and IV of the spinal cord gray matter; LH, lateral hypothalamic nucleus; NTS, nucleus of the tractus solitaries; PVH, paraventricular hypothalamic nucleus; RVLM, rostral ventrolateral medulla; T6, spinal cord thoracic 6 segment.

Parasympathetic motor innervation of the pancreas consists of preganglionic neurons in the DMV and peripheral postganglionic neurons (intramural ganglia) located in the pancreas (Rinaman and Miselis, 1987; Figure 2). Tracing studies suggest that the pancreatic parasympathetic preganglionic neurons receive input from the ventromedial medulla, the medullary raphe region, the medullary reticular area, the pontine A5 area, the hypothalamus, the subfornical organ, the bed nucleus of stria terminalis, and the cortex (Loewy and Haxhiu, 1993; Loewy et al., 1994; Streefland et al., 1998; Buijs et al., 2001).

The direct effects of DMV on pancreatic endocrine and exocrine secretions have been demonstrated. Mussa and colleagues have made recordings from pancreas-projecting preganglionic vagal motor neurons and found their fibers had axonal conduction velocities in the C-fiber range (Mussa et al., 2010; Mussa and Verberne, 2013). Furthermore, they showed that activation of the DMV by bilateral injection of bicuculline methiodide (GABA A receptor antagonist) induces a rapid elevation in glucose-induced insulin secretion *via* a cholinergic mechanism, while the inhibition of this pathway decreases the pancreatic insulin secretion (Mussa et al., 2011). Activation of the DMV also leads to increases in the pancreatic exocrine secretion and pancreatic protein output through a cholinergic muscarinic mechanism (Mussa and Verberne, 2008).

Interestingly, DMV activity is known to be regulated by the pancreatic polypeptide- (e.g., NPY and peptide YY) mediated vago-vagal reflex (Chen and Rogers, 1997; Browning and Travagli, 2003). In one of their elegant studies, Browning and Travagli used whole-cell patch clamp recordings from the brainstem slice DMV neurons to demonstrate that NPY and peptide YY inhibit the excitatory synaptic transmission between NTS and DMV *via* the NPY family receptor-dependent mechanism (Browning and Travagli, 2003). It is worth mentioning that the functions of the pancreatic endocrine and exocrine are regulated by distinct DMV neurocircuits (Babic and Travagli, 2014). The vagal control of the pancreatic endocrine or exocrine secretions depends on the firing rate of DMV neurons (Berthoud and Powley, 1987).

Pancreas-specific sympathetic motor innervations have been characterized in the spinal cord. However, the projections from the higher-order brain regions to these neurons are less clear. Tracing studies indicate brain areas that appear to overlap with the parasympathetic motor innervation including the ventromedial medulla, the medullary raphe region, the medullary reticular area, the pontine A5 area, and the hypothalamic nuclei (Berthoud and Powley, 1996; Jansen et al., 1997; Buijs et al., 2001). Results from developmental studies suggest that sympathetic innervation is necessary for pancreatic islet architecture and functional maturation during embryogenesis (Borden et al., 2013; Reinert et al., 2014).

### Motor Innervation

#### Motor Efferent Fibers of the Vagus Nerve (Parasympathetic Efferent Pathway)

Motor efferent fibers of the vagus nerve originate predominantly in the left medial and lateral cell column of the DMV (Luiten et al., 1984; Sharkey et al., 1984; Fox and Powley, 1985; Rosario et al., 2016; Kenny and Bordoni, 2020). Relatively sparse fibers originate in the nucleus ambiguus that is located ventral, lateral, and slightly rostral to the DMV. These neurons are heterogeneous in size and excitability, different from other gastrointestinal organ-projecting neurons which are more homogeneous in these characteristics (Browning et al., 2005a,b). After passing through the hiatus of the esophagus, the vagus nerve fibers divide into five branches (i.e., hepatic, anterior and posterior gastric, and anterior and posterior celiac) in the abdomen (Fox and Powley, 1985). The parasympathetic efferent nerves innervating the pancreas travel in the same vagus nerve sheath along with the parasympathetic afferent nerves mostly through the hepatic and bilateral gastric branches and a few collaterals from the bilateral celiac branches (Chandra and Liddle, 2014; Li et al., 2019). In agreement with these observations, it has been shown that the hepatic and gastric branches, but not the celiac branches are responsible for insulin and glucagon secretion (Berthoud and Powley, 1990).

Fibers of the parasympathetic preganglionic neurons terminate to synapses with nicotinic receptors that are primarily expressed on the intrapancreatic ganglionic neurons, as well as to the sympathetic efferent fibers, islets, and vasculature. Postganglionic parasympathetic fibers from the intrapancreatic ganglia reach islets, acini, and vasculature expressing the muscarinic type 1 and 3 acetylcholine receptors (Rodriguez-Diaz et al., 2011, 2012). Acetylcholine released by postganglionic parasympathetic nerve fibers can stimulate glucagon-secreting α-cells, insulin-secreting β-cells, somatostatin-secreting δ-cells, exocrine acinar cells, and epithelia of the duct system (Love et al., 2007; Rodriguez-Diaz et al., 2011; Molina et al., 2014). Moreover, it has been shown that group II metabotropic glutamate receptors regulate both pancreatic exocrine and insulin secretions, whereas group III metabotropic glutamate receptors only control insulin release (Browning and Travagli, 2007; Travagli and Anselmi, 2016). In addition to the direct regulation from DMV, Owyang and Logsdon have documented that cholecystokinins released from enteroendocrine cells of the small intestine interact with cholecystokinin-A receptors on vagal afferents to modulate exocrine secretion (Owyang and Logsdon, 2004). Glucagon-like peptide-1 can increase pancreatic insulin secretion *via* a direct action on pancreatic β-cells and indirectly by acting on DMV neurons to control pancreatic endocrine secretion (Thorens, 1995; Wan et al., 2007; Travagli and Anselmi, 2016).

The postganglionic parasympathetic nerve fibers also release vasoactive intestinal polypeptide, gastrin-releasing peptide, pituitary adenylate cyclase-activating polypeptide, NPY, galanin, and nitric oxide (NO; Pettersson et al., 1987; Love et al., 2007; Di Cairano et al., 2016; Li et al., 2019). Among them, the former three are known to stimulate and potentiate the secretion of glucagon and insulin (Bertrand et al., 1996; Filipsson et al., 2001; Di Cairano et al., 2016). NPY and galanin exist in both parasympathetic and sympathetic nerve fibers of the pancreas. Although their effects remain controversial and are species-specific, most reports suggest they negatively mediate pancreatic endocrine and exocrine secretions (Love et al., 2007; Di Cairano et al., 2016; Li et al., 2019). A novel role of NPY in stimulating β-cell proliferation and survival has also been suggested (Cho and Kim, 2004). Finally, an inhibitory role for NO in insulin secretion has been demonstrated *in vitro* and *in vivo*. Supplementing isolated islets with NO synthesis inhibitor enhances glucose-induced insulin secretory response while treating these cells with NO donor hydroxylamine suppresses insulin release (Salehi et al., 1996). Similar observations were also evident *in vivo*. Treating mice with NO synthesis inhibitor systemically also potentiates the DMV-mediated glucose-induced insulin secretion (Mussa et al., 2011). It has been postulated that NO may inhibit the release of acetylcholine and subsequently insulin release (Wang et al., 1999; Mussa et al., 2011).

#### Motor Efferent Fibers of the Sympathetic Nerve (Sympathetic Efferent Pathway)

The sympathetic motor innervation of the pancreas consists of preganglionic and postganglionic neurons. The cell bodies of the preganglionic efferent fibers are located in the intermediolateral columns of the spinal cord at T6–L2 segmental levels (Rinaman and Miselis, 1987; Chen et al., 1996; Furuzawa et al., 1996; Jansen et al., 1997; Buijs et al., 2001; Quinson et al., 2001). Some fibers pass the paravertebral ganglia without forming a synapse and travel *via* the splanchnic nerves to form synapses within the celiac ganglia and the superior mesenteric ganglion (Dolenšek et al., 2015; Li et al., 2019); whereas, some postganglionic neurons are located in the paravertebral ganglia of the sympathetic chain (Figure 2). Postganglionic sympathetic fibers accompany branches of the celiac trunk (e.g., splenic, pancreatic, and pancreaticoduodenal arteries) to innervate vasculature, islets, acini, and intrapancreatic ganglia (Alm et al., 1967; Carlei et al., 1985; Su et al., 1987; Niebergall-Roth and Singer, 2001; Yi et al., 2004; Rodriguez-Diaz and Caicedo, 2014).

Preganglionic sympathetic fibers are cholinergic, whereas postganglionic sympathetic fibers are predominantly noradrenergic with minor secretions of NPY and galanin. Sympathetic nerves inhibit insulin secretion, decrease blood flow, and inhibit digestive enzyme production by inhibiting parasympathetic postganglionic innervation (Ahrén et al., 1987a,b; Holst et al., 1993; Niebergall-Roth and Singer, 2001; Dolenšek et al., 2015; Morton et al., 2017). In contrast, it has been suggested that parasympathetic preganglionic fibers inhibit pancreatic sympathetic nerves (Benthem et al., 2001).

## Intrapancreatic Nervous Division

**Figure 3:**
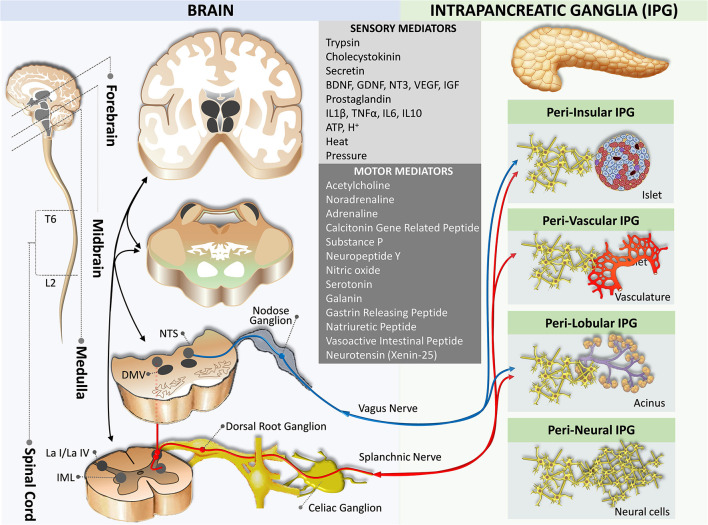
Neural crosstalk between extrinsic motor innervation from the brain and the intrapancreatic ganglia. The blue pathway marks the extrinsic parasympathetic efferent pathway, while the red pathway indicates the extrinsic sympathetic efferent pathway. DMV, dorsal motor nucleus of the vagus; IML, intermediolateral nucleus of the spinal cord; L2, spinal cord lumbar 2 segment; La I/La IV, lamina I and IV of the spinal cord gray matter; NTS, nucleus of the tractus solitaries; T6, spinal cord thoracic 6 segment.

The association between neurons and endocrine cells of pancreatic islets (also termed neuro-insular complex) has been studied in a variety of mammals. Two types of the neuro-insular complex have been proposed: (I) a gathering of islet cells and ganglionic cells; and (II) bundles of nerve fibers in close contact with islet cells (Proshchina et al., 2014). Intrapancreatic ganglia are frequently found alongside nerve trunks in the interlobular, acinar, and within lobules and islets (Kirchgessner and Pintar, 1991; Tang et al., 2018a; Figure 3). The majority of neurons in the intrapancreatic ganglia are cholinergic. However, they also synthesize a large number of biologically active substances including NPY, CGRP, NO, vasoactive intestinal polypeptide, norepinephrine, enkephalin, and gastrin-releasing peptide, which resemble neurons in the myenteric plexus of the enteric nervous system of the gut but differ by excitability (Larsson and Rehfeld, 1979; Larsson, 1979; De Giorgio et al., 1992; Esteves et al., 1993; Shimosegawa et al., 1993; Hiramatsu and Ohshima, 1994; Liu et al., 1998; Yi et al., 2004). Intrapancreatic ganglia are more abundant in the perivascular plexus and fewer in the perineural plexus (Liu and Kirchgessner, 1997; Love and Szebeni, 1999; Krivova et al., 2016).

Although these intrapancreatic neurons are considered postganglionic parasympathetic neurons, the role of these neurons in the endocrine and exocrine functions of the pancreas is not well understood. Receptors for autonomic neurotransmitters are expressed on the plasma membrane of islet cells, and it is known that these neurotransmitters modulate islet hormone secretion (Ahrén, 2000). In addition to innervating blood vessels, autonomic axons in the mouse islet penetrate the parenchyma to establish contacts with endocrine cells (Rodriguez-Diaz and Caicedo, 2014).

## Enteropancreatic Plexus

The gut is innervated by both the sympathetic and parasympathetic nervous systems as well as by the primary sensory nerves. The densest and most widespread innervations are derived from vagal afferent neurons located in the nodose ganglia and their proximal extensions terminate in the NTS (Berthoud et al., 1997). Sensory terminals innervating both the myenteric plexus and the submucous plexus of the gut act as mechanosensors and chemosensors, respectively (Blackshaw and Grundy, 1990; Berthoud and Powley, 1992; Berthoud and Patterson, 1996; Zagorodnyuk et al., 2001; Powley et al., 2011).

**Figure 4:**
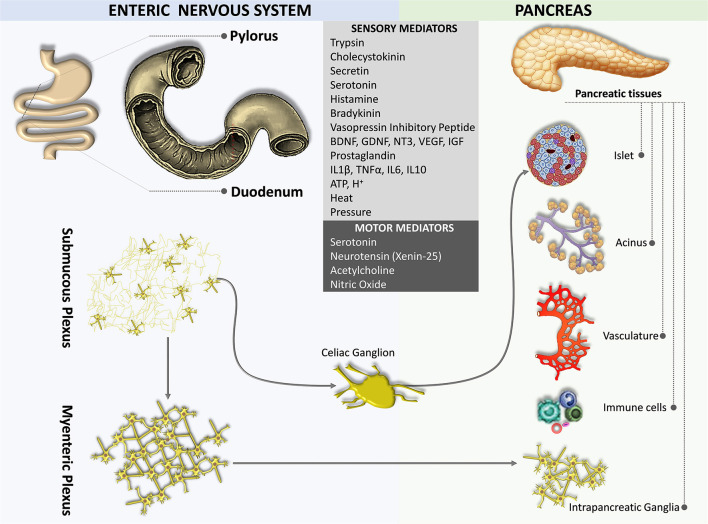
Neural crosstalk between the pancreas and the gut.

An enteropancreatic innervation has been demonstrated. Tracing studies in rodents suggest that neurons in the myenteric plexus of the antrum of the stomach and the initial part of the duodenum sent axons to the pancreas terminated in the intrapancreatic ganglia, although some fibers were also observed near acini, ducts, vessels, and islet cells (Kirchgessner and Gershon, 1990, 1995; Kirchgessner et al., 1996; Chandrasekharan and Srinivasan, 2007; Figure 4). Some of the enteropancreatic neurons are cholinergic, which form excitatory nicotinic synapses on neurons in intrapancreatic ganglia (Kirchgessner and Gershon, 1995; Kirchgessner and Liu, 2001). Stimulation of neurons in the myenteric plexus of the duodenum leads to the activation of neurons in intrapancreatic ganglia (Kirchgessner et al., 1996). Enteropancreatic innervations with 5-HT immunoreactive axons have also been identified (Kirchgessner and Gershon, 1990). These 5-HT axons are believed to form inhibitory axo-axonic synapses in the pancreas (Kirchgessner and Gershon, 1995; Li et al., 2019). Also, some enteropancreatic nerves contain the pituitary adenylate cyclase-activating peptides, which are often considered as neuromodulators that strengthen pancreatic secretion (Kirchgessner and Liu, 2001; Li et al., 2019).

## Humoral Crosstalk

**Figure 5:**
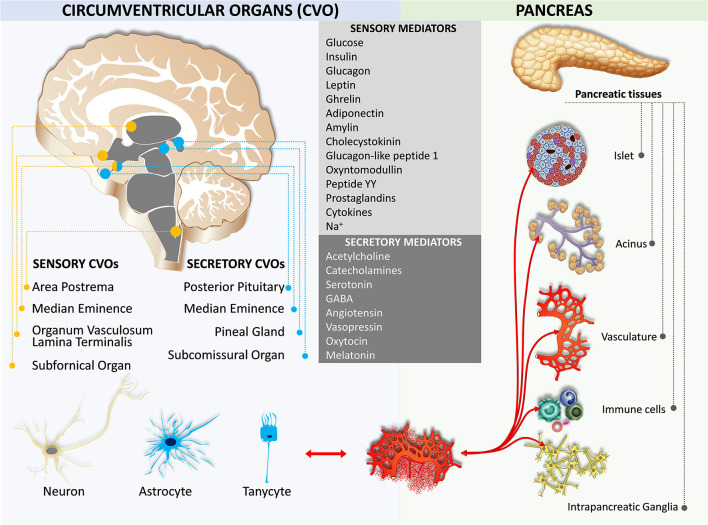
Humoral crosstalk between the circumventricular organs and the pancreas.

Early studies on feeding behavior indicated the role of hypothalamic nuclei in metabolic homeostasis (Brobeck, 1946; Kennedy, 1966). Later, leptin, an adipocyte-derived hormone, is exhibited to decrease body weight through the leptin receptor in the circumventricular organ of the brain (Maffei et al., 1995; Ahima, 2008). There are seven circumventricular organs including the area postrema, the vascular organ of the lamina terminalis, the subfornical organ, the median eminence, the subcomissural organ, the pineal gland, and the posterior pituitary (Cottrell and Ferguson, 2004; Miyata, 2015; Figure 5). The first three organs contain neurons that are capable of detecting circulating mediators due to the lack of a functional blood-brain barrier (Johnson and Gross, 1993). The other four organs secrete hormones and do not have a blood-brain barrier, except the subcomissural organ that has a fully functional blood-brain barrier (Miyata and Hatton, 2002). Notably, the median eminence is both a secretory and a sensory organ. It has been shown that tanycytes in the median eminence express a variety of receptors for sensing nutrient signals, including leptin, insulin-like growth factor-1, nerve growth factor, corticotropin-releasing factor, and thyroid-stimulating hormone (Bohannon et al., 1986; Werther et al., 1989; Yan and Johnson, 1989; Potter et al., 1994; Banks et al., 1996; Rodríguez et al., 2010; Lee et al., 2012; Bolborea et al., 2015).

### Sensory Mediators

The area postrema located on the caudal brainstem integrates circulating metabolic signals including leptin, amylin, cholecystokinin, glucagon-like peptide-1, peptide YY, and ghrelin which affect energy and metabolic homeostasis (Fenstermacher et al., 1988; Fry et al., 2007). This information is transmitted to the adjacent NTS which in turn relays to other brain areas including several hypothalamic nuclei (Grill and Hayes, 2012). Area postrema neurons project to the pancreas-projecting DMV neurons and are responsible for basal and vago-vagal reflex stimulated pancreatic enzymes (Deng et al., 2001a,b, c; Browning et al., 2005a,b). Insulin and amylin secreted from β cells, glucagon secreted from the α cells, somatostatin secreted from the δ cells, pancreatic polypeptide secreted from the γ cells, glucagon-like peptide produced from the small intestine, and ghrelin secreted from the gastric cells and pancreatic ε cells are involved in the hypothalamic and medullary circuits that affect both parasympathetic and sympathetic innervation of the pancreas *via* humoral pathways (Taylor et al., 1979; Whitcomb et al., 1990; Ying and Chung, 1993; Okumura Y. et al., 1995; Orskov et al., 1996; Wettergren et al., 1998; Unniappan and Kieffer, 2008; Trevaskis et al., 2010; Clemmensen et al., 2014). Thyrotropin-releasing hormone secreted from both the hypothalamus and islets stimulate digestive enzyme secretion (Kato and Kanno, 1983; Messmer et al., 1993; Okumura T. et al., 1995).

### Secretory Mediators

The posterior pituitary secretes two important secretory hormones, arginine vasopressin (AVP) and oxytocin. Magnocellular AVP- and oxytocin-containing neurons are mainly located in the paraventricular nucleus and supraoptic nuclei of the hypothalamus. A few accessory cells can also be found in the medial and lateral hypothalamus (Cunningham and Sawchenko, 1991; Qin et al., 2018). It has been shown that the serotonergic control of AVP and oxytocin secretion from the neurohypophyseal tissue in rats was observed at the level of the posterior pituitary (Gálfi et al., 2005). The magnocellular AVP neurons release AVP from their dendrites (Brownstein et al., 1980; Ludwig and Leng, 2006). Some parvocellular AVP neurons also synthesize the corticotropin-releasing factor. These AVP neurons mainly project to the median eminence, from which it reaches the anterior pituitary gland to regulate the secretion of adrenocorticotropic hormone upon stress stimulation (Ludwig and Leng, 2006). Interestingly, the magnocellular neurons project to the brainstem and spinal cord to be involved in the mechanism of glucocorticoid escape (Chandra and Liddle, 2009; Antoni, 2019).

Oxytocin neurons in the paraventricular and supraoptic nuclei project to the arcuate nucleus. Oxytocin neurons in the paraventricular nucleus also project to the dorsal vagal complex, including the NTS, the area postrema, and the DMV to inhibit control of the vagal preganglionic neurons that innervate the pancreas (Siaud et al., 1991; Maejima et al., 2018). Recent studies have identified the effects of oxytocin feeding reward and the regulation of homeostatic feeding (Maejima et al., 2018). The mechanisms of oxytocin in food intake regulation have also been studied. It has been shown that central insulin action induced activation of paraventricular nucleus magnocellular oxytocin neurons to release oxytocin into circulation, possibly serving as a mechanism for the regulation of energy and metabolic homeostasis (Zhang et al., 2018).

## Neural Regulation of Blood Glucose Level

Pancreatic islet *α*- and *β*-cells are regulated by glucose concentrations, with hypoglycemia-triggering *α*-cells release glucagon and hyperglycemia-stimulating *β*-cells secret insulin. There are other specialized glucose-sensing cells located in the periphery (e.g., the hepatoportal vein area) and the central nervous system (e.g., the hypothalamus, the dorsal vagal complex, and the basolateral medulla areas; Thorens, 1995, 2011). The peripheral and central glucose-sensing cells/neurons form a network monitoring systemic blood glucose concentrations. This reciprocal network, after integration with aforementioned sensory afferent inputs and humoral mediator-elicited signals, calibrates autonomic tone to global homeostatic need by adjusting functions of the pancreas along with other organs/systems. Both divisions of the autonomic nervous system are known to affect the islets at multiple aspects, including the proliferation and maturation of the islets and the control of insulin secretion, in particular the cephalic phase (Ahrén, 2000; Thorens, 2011, 2014). Their roles in the regulation of islet cells are briefly described here:

### Parasympathetic Division

The parasympathetic innervation of the islets potentiates insulin secretion during hyperglycemia through a cholinergic muscarinic mechanism (Mussa and Verberne, 2008; Rodriguez-Diaz et al., 2011). Neuropeptides and NO found in parasympathetic nerve terminals also contribute to the parasympathetic control of β-cells (Thorens, 1995; Wang et al., 1999; Wan et al., 2007; Mussa et al., 2011; Travagli and Anselmi, 2016). Parasympathetic innervation of the islets is involved in the control of the cephalic phase of insulin secretion (Berthoud and Powley, 1990). Parasympathetic activity is increased during hypoglycemia to stimulate glucagon secretion and subsequent restoration of euglycemia (Taborsky and Mundinger, 2012). However, it is worthy to note that the effect of parasympathetic innervation on blood glucose homeostasis seems to depend on innervation densities and human islets are less innervated by the parasympathetic fibers than mouse islets (Rodriguez-Diaz et al., 2011). Furthermore, the distributions of islet-acinar neurovascular association between humans and mice are fundamentally different (Tang et al., 2018b). Hence, whether the results of parasympathetic activity on the regulation of blood glucose derived from rodent studies can be translated to humans deserves further in-depth inspection.

### Sympathetic Division

The sympathetic nervous system plays significant role in regulating both α- and β-cell function. By examining human pancreases *via* 3-dimensional panoramic histology with tissue clearing, sympathetic innervation of human islets was found on smooth muscle cells of the intra-islet vasculature and α-cells (Tang et al., 2018b). Norepinephrine, released from postganglionic sympathetic fibers and adrenal glands, stimulates glucagon secretion *via* binding to β2-adrenergic receptors on the α-cells but inhibits insulin secretion *via* binding to α2-adrenergic receptors on the β-cells (Ahrén et al., 1987a,b; Holst et al., 1993; Niebergall-Roth and Singer, 2001; Dolenšek et al., 2015; Morton et al., 2017). Overexpression of the α2-adrenergic receptor on the β-cells is known to impair insulin granule docking at the plasma membrane, reduce β-cell exocytosis and insulin secretion, and increase type 2 diabetes risk (Rosengren et al., 2010).

Sympathetic innervation is critical for the formation of the pancreatic islets and their functional maturation, while deprivation of sympathetic innervation during development leads to abnormal islet architecture, reduced insulin secretion, and impaired glucose tolerance in adult mice (Borden et al., 2013). Furthermore, a loss of sympathetic efferents to the pancreas is associated with the pathogenesis of metabolic disorders (Borden et al., 2013). Individuals with spinal cord injury are more likely to have metabolic abnormalities that are associated with higher morbidity rates of diabetes and higher rates of diabetes-related complications and mortality (Cragg et al., 2013; Gorgey et al., 2014). Spinal cord injury can induce fat accumulation, physical inactivity, and body composition changes resulting in insulin resistance and glucose intolerance, which eventually leads to type 2 diabetes (Bauman and Spungen, 2000; Gorgey et al., 2014).

### Hypoglycemia-Associated Autonomic Failure

The role of neural regulation of blood glucose level is particularly evident in hypoglycemia-associated autonomic failure in diabetes, a serious threat to diabetic patients with insulin treatment (Cryer, 2006). Intensive treatment of patients with diabetes can initiate a hypoglycemic episode. Normal physiological responses to hypoglycemia mainly comprise activation of the sympathetic nervous system, secretions of norepinephrine from the sympathetic postganglionic nerve terminals, epinephrine from the adrenal medulla, glucagon from the pancreas, cortisol from the adrenal cortex, and growth hormones from the pituitary gland (Diedrich et al., 2002; Rickels, 2019). However, diabetic patients with repeated hypoglycemic episodes may display a progressive deficiency in their ability to respond to hypoglycemia by inappropriate secretion of glucagon and other counterregulatory hormones such as epinephrine, cortisol, and growth hormones (Cryer, 2013). Furthermore, some patients may lose recognition of hypoglycemic symptoms, mainly due to blunted sympathetic responses. This condition is termed hypoglycemia-associated autonomic failure in diabetes (Dagogo-Jack et al., 1993; Cryer, 2013). The blunted sympathetic/sympathoadrenal responses evident in patients with hypoglycemia-associated autonomic failure cannot be fully explained by diabetic autonomic neuropathy, since the blunted sympathetic/sympathoadrenal responses can be induced in nondiabetic persons and can be reversed in patients with diabetes (Dagogo-Jack et al., 1993; Arbelaez et al., 2008). Delineation of biological and molecular mechanisms underlying the blunted sympathetic/sympathoadrenal responses to hypoglycemia that cause hypoglycemia-associated autonomic failure is still an active research topic today.

## The Advantages and Disadvantages of The Methods for Pancreas Innervation Identification

This review refers to several methods used to identify the innervation of the pancreas. However, caution must be taken when accepting these findings as some of the methods may exaggerate the distribution of the innervation. For example, it has been shown that some of the tracing studies using conventional retrograde tracers overestimated the number of parasympathetic preganglionic neurons that project monosynaptically to the pancreas and their distribution in the medulla possibly due to diffusion of the tracer to adjacent abdominal organs (Fox and Powley, 1986). Similar problems are likely to also occur with the use of neurotropic viral tracers (Streefland et al., 1998; Buijs et al., 2001). It has been suggested that microinjection of the anterograde tracers into the DMV followed by identification of labeled fibers in the pancreas is a more convincible approach to detect the efferent innervation of the pancreas (Berthoud and Powley, 1991).

## Conclusion

The endocrine and exocrine functions of the pancreas are regulated tightly by the complex and integrated responses of the functional nervous circuits. Sensory fibers of both parasympathetic and sympathetic pathways terminate monosynaptically in the vagus nerve centers and the dorsal horn neurons at T6-L2 segmental levels of the spinal cord, respectively. Medullary neurons in the NTS, the DMV, and the area postrema form a functional spino-medullary circuit for somato-autonomic reflexes to modulate both the endocrine and exocrine functions in the pancreas polysynaptically. The reticular activating system, mesencephalic homeostatic centers, the hypothalamic autonomic nervous system, thalamocortical circuits, and interoceptive centers play modulatory roles in the regulation of pancreatic functions.

## Author Contributions

The first draft of the manuscript was written by BL and all authors contributed to editorial changes in the manuscript. All authors contributed to the article and approved the submitted version.

## Conflict of Interest

The authors declare that the research was conducted in the absence of any commercial or financial relationships that could be construed as a potential conflict of interest.
